# Flavored and Nicotine-Containing E-Cigarettes Induce Impaired Angiogenesis and Diabetic Wound Healing via Increased Endothelial Oxidative Stress and Reduced NO Bioavailability

**DOI:** 10.3390/antiox11050904

**Published:** 2022-05-05

**Authors:** Zhuoying Liu, Yixuan Zhang, Ji Youn Youn, Yabing Zhang, Ayako Makino, Jason X.-J. Yuan, Hua Cai

**Affiliations:** 1 Department of Anesthesiology, Department of Medicine/Cardiology, David Geffen School of Medicine, University of California Los Angeles, Los Angeles, CA 90095, USA; zuoyingliu@ucla.edu (Z.L.); yixuanzhang@ucla.edu (Y.Z.); jyyoun@ucla.edu (J.Y.Y.); yabingzhang@ucla.edu (Y.Z.); 2 Department of Medicine, University of California San Diego, San Diego, CA 92093, USA; amakino@health.ucsd.edu (A.M.); jxyuan@health.ucsd.edu (J.X.-J.Y.)

**Keywords:** e-cigarettes (nicotine containing and flavored), endothelial dysfunction, oxidative stress, nitric oxide (NO) deficiency, apoptosis, angiogenesis, diabetic wound healing

## Abstract

The prevalent use of electronic cigarettes (e-cigarettes) has increased exponentially in recent years, especially in youth who are attracted to flavored e-cigarettes. Indeed, e-cigarette or vaping product use-associated lung injury (EVALI) cases started to emerge in the United States in August 2019, resulting in 2807 hospitalized cases and 68 deaths as of 18 February 2020. In the present study, we investigated, for the first time, whether flavored and nicotine containing e-cigarettes induce endothelial dysfunction to result in impaired angiogenesis and wound healing particularly under diabetic condition. Nicotine containing e-cigarettes with various contents of nicotine (0, 1.2%, 2.4%), and flavored e-cigarettes of classic tobacco, mint, menthol, and vanilla or fruit from BLU (nicotine 2.4%) or JUUL (nicotine 3%), were used to treat endothelial cells in vitro and streptozotocin-induced diabetic mice in vivo. Endothelial cell superoxide production, determined by dihydroethidium (DHE) fluorescent imaging and electron spin resonance (ESR), was markedly increased by exposure to e-cigarette extract (e-CSE) in a nicotine-content dependent manner, while nitric oxide (NO) bioavailability detected by DAF-FM fluorescent imaging was substantially decreased. All of the different flavored e-cigarettes examined also showed significant effects in increasing superoxide production while diminishing NO bioavailability. Endothelial cell apoptosis evaluated by caspase 3 activity was markedly increased by exposure to e-CSE prepared from flavored and nicotine containing e-cigarettes. Endothelial monolayer wound assays revealed that nicotine-containing and flavored e-cigarettes induced impaired angiogenic wound repair of endothelial cell monolayers. Furthermore, vascular endothelial growth factor (VEGF) stimulated wound healing in diabetic mice was impaired by exposure to e-CSEs prepared from nicotine-containing and flavored e-cigarettes. Taken together, our data demonstrate for the first time that flavored and nicotine-containing e-cigarettes induce endothelial dysfunction through excessive ROS production, resulting in decreased NO bioavailability, increased endothelial cell apoptosis, and impairment in angiogenesis and wound healing, especially under diabetic condition. These responses of endothelial dysfunction likely underlie harmful effects of e-cigarettes in endothelial-rich organs, such as heart and lungs. These data also indicate that rigorous regulation on e-cigarette use should be enforced in diabetic and/or surgical patients to avoid severe consequences from impaired angiogenesis/wound healing.

## 1. Introduction

E-cigarettes are electronic nicotine delivery systems (ENDS) originally designed for smoking cessation [1]; however, their effect on smoking cessation has not been proven. Rather, their use has turned out to be a new, normalized pattern of smoking since the majority of the e-cigarettes contain nicotine and other toxic components beyond those in regular tobacco products [2]. The number of e-cigarette users has increased exponentially in recent years, while the toxic effects of e-cigarette have also become evident [3]. In 2018, the US Surgeon General and Food and Drug Administration (FDA) officially announced teenage use of e-cigarettes an epidemic. According to a national youth tobacco survey 2021, more than 2 million US teenagers, including 1.72 million high school students (11.3%) and 0.32 million middle school students (2.8%), are current e-cigarette users. Of concern, the percentage of users of flavored e-cigarettes among all e-cigarette users has increased from 65.1% to 84.7% from 2014 to 2020, and that various fashionable flavors, such as fruit, mint, and menthol, have been shown to be particularly appealing to youth [4]. Not just that the flavoring additives have toxic effects, the flavored e-cigarettes also contain nicotine, with it being known that vaporized nicotine and solvent decomposition products can induce oxidative tissue injuries [5].

Notably, e-cigarette or vaping product use-associated lung injury (EVALI) cases started to emerge in the United States in August 2019, resulting in 2807 hospitalized cases and 68 deaths as of 18 February 2020 [6]. While the affected patients were 13–85 years old, more than half of them were teenagers (15%, <18 years old) or young adults (37%, 18–24 years old) with excessive use of flavored and nicotine-containing e-cigarettes. However, underlying mechanisms for EVALI remain elusive. The key pathological condition of EVALI is acute lung injury characterized by lung inflammation and organizing pneumonia potentially linked to endothelial dysfunction [7]. In particular, exposure to e-cigarettes has been shown to impair mucociliary function by reducing airway surface liquid volume, ciliary beat frequency, and transepithelial ion transport so that mucus clearance is disrupted [8]. The alterations in alveolar macrophage functions by exposure to e-cigarettes can lead to lung inflammation and tissue damages [9]. In addition to respiratory consequences, exposure to e-cigarettes has been shown from clinical trials to exert toxic effects on the cardiovascular system [10,11]. Acute exposure to nicotine containing e-cigarettes results in an increase in mean arterial pressure while it decreases muscle sympathetic nerve activity in healthy young non-smokers [10]. Similarly, acute use of e-cigarettes in young adults was found to be associated with higher perturbations in heart rate variability, and increased plasma oxidized markers assessed by LDL oxidizability, HDL antioxidant index, and paraoxonase-1 activity [11]. In mice exposed to e-cigarettes for 60 weeks, there were significant elevations in blood pressure, cardiac hypertrophy, and aortic thickening, accompanied by impaired endothelium dependent and independent vasodilation, to similar extents as in tobacco cigarette-exposed mice [12]. This evidence seems to suggest that use of e-cigarettes may indeed predispose subjects to cardiovascular diseases. Nonetheless, acute and chronic effects of e-cigarettes on endothelial function and its underlying mechanisms have not been fully elucidated.

Therefore, we aimed to examine, for the first time, specific effects of flavored and nicotine-containing e-cigarettes on endothelial nitric oxide (NO) bioavailability and endothelial cell apoptosis attributed to a potential increase in reactive oxygen species (ROS) production, as well as effects of e-cigarettes on angiogenesis and diabetic wound healing that requires sufficiently available NO to drive coordinated endothelial cell proliferation and migration. Our data indicate that flavored and nicotine-containing e-cigarettes induce endothelial dysfunction through excessive production of ROS, and consequent endothelial apoptosis and reduction in NO bioavailability, resulting in impaired angiogenesis and diminished wound healing especially under diabetic condition.

## 2. Methods

### 2.1. Cell Culture

Bovine aortic endothelial cells (BAECs, Gelantis, passages 3–7) were cultured in M199 media (MT10060CV, Thermo Fisher Scientific, Waltham, MA, USA) supplemented with 10% fetal bovine serum (FBS, MT35010C, Thermo Fisher Scientific, Waltham, MA, USA), 1% vitamins (MT25020CI, Thermo Fisher Scientific, Waltham, MA, USA), 1% L-glutamine (25030081, Thermo Fisher Scientific, Waltham, MA, USA), and Penicillin–Streptomycin (15140122, Thermo Fisher Scientific, Waltham, MA, USA) until confluence as previously described [13,14,15,16,17,18]. The FBS present in the media was lowered to 5% to starve the cells prior to treatments.

### 2.2. E-Cigarettes Used

E-cigarettes from two major vendors were used for the present study, including e-cigarettes containing various nicotine concentrations from Blu (Charlotte, NC, USA), Blu 0% nicotine (Classic Goldleaf, I2149321), Blu 1.2% nicotine (Classic Goldleaf, I2149319), and Blu 2.4% nicotine (Classic Goldleaf, I2149320); flavored e-cigarettes from Blu, Blu Classic 2.4% nicotine (I2149320), Blu Mint 2.4% nicotine (I2149059), Blu Menthol 2.4% nicotine (I7001B), and Blu Vanilla 2.4% nicotine (I7005B); flavored e-cigarettes from JUUL (San Francisco, CA, USA), JUUL Classic 3.0% nicotine, JUUL Mint 3.0% nicotine, JUUL Menthol 3.0% nicotine, and JUUL Fruit 3.0% nicotine (Note that these products from JUUL do not have specific catalog numbers).

### 2.3. Preparation of E-CSE and Exposure to Endothelial Cells

E-cigarette smoke extract (e-CSE) was prepared by applying a vacuum force to a beaker that draws 350 mL of water from the second beaker during the preparation process; the vacuum created from the second beaker draws smoke into a Falcon tube containing cell culture medium from attached e-cigarettes (either Blu or JUUL) 10 times with a 2 s break each time to simulate human use; the preparation is considered a 100% stock. One day post confluent endothelial cells were exposed to e-CSE prepared from e-cigarettes containing different concentrations of nicotine and flavoring additives (see above) by directly adding e-CSE to the cell culture media with the final concentration of 10% for e-CSE.

### 2.4. Determination of Superoxide Production Using Electron Spin Resonance (ESR)

Confluent BAECs were starved overnight in M199 medium containing 5% FBS, and incubated with 10% of e-CSE for 24 h. Superoxide production was measured using electron spin resonance (ESR) (eScan, Bruker, Billerica, MA, USA) as we previously described [15,18,19]. Briefly, BAECs were washed, and cell pellets collected in cold KHB buffer. Superoxide-specific spin trap methoxycarbonyl-2,2,5,5-tetramethyl-pyrrolidine (CMH, 1 mmol/L, ALX-430-117-M250, Enzo Life Sciences, Framingdale, NY, USA) was prepared in nitrogen gas bubbled KHB buffer containing diethyldithiocarbamic acid (5 μmol/L, D3506, MilliporeSigma, St. Louis, MO, USA) and deferoxamine (25 μmol/L; D9533, MilliporeSigma, St. Louis, MO, USA). Resuspended cells were mixed with spin trap in the presence of water (baseline) or PEG-SOD (polyethylene glycol-superoxide dismutase; 20 U/mL; S9549, MilliporeSigma, St. Louis, MO, USA), and loaded immediately into ESR spectrometer. The PEG-SOD inhibitable signals were used to calculate superoxide production and normalized to protein concentrations.

### 2.5. Endothelial Cell Monolayer Wound Closure Assay

Endothelial cell monolayer wound assay was performed as we previously published [13]. BAECs were cultured on 35 mm dishes. Cells were allowed to grow till confluence, and then starved overnight in M199 medium containing 5% FBS. The 1-mm scratch wounds were created with a P-1000 tip on confluent endothelial monolayers, followed by washing with phosphoate-buffered saline (PBS) to remove debris. Images of the scratch were captured at the locations marked at the bottom of the culture dish, which is considered as t = 0 h. The cells were then treated with indicated e-CSEs prepared from flavored e-cigarettes or e-cigarettes containing different amounts of nicotine. After incubation for 24 h, images were taken at the same locations. Wound areas were analyzed by NIH ImageJ software (given as numbers of pixels). Area of migration was calculated by subtracting area measured at 0 h from area measured at 24 h.

### 2.6. Wound Healing Assay in Diabetic Mice

Male C57BL/6 mice (10–12 weeks old) were purchased from Charles River Laboratories (Wilmington, MA, USA). The use of animals and experimental procedures were approved by the Institutional Animal Care and Usage Committee at the University of California, Los Angeles (UCLA). Wound healing assay in diabetic mice was carried out as we previously published [16]. Wild type male C57BL/6 mice (10–12 weeks old) were treated with two injections (i.p.) of streptozocin (STZ, 100 mg/kg, S0130, MilliporeSigma St. Louis, MO, USA) [16,19]. Blood glucose levels were measured with a OneTouch Ultra blood glucose meter (LifeScan, Malvern, PA, USA) to confirm successful induction of diabetes. The animals were then anesthetized and shaved at the dorsum area. On day 0, the skin was prepared with Betadine and 70% ethanol before a full thickness of 6-mm skin punch biopsy (Miltex 33–36, Integra LifeSciences, Plainsboro, NJ, USA) was made. The wounds were treated every other day with recombinant human VEGF (200 ng, 293-VE-010, R&D system), and 5 μL of e-CSE stock and allowed to dry. Control mice were treated with 5 μL of PBS in the same fashion. Then, a bioclusive transparent dressing (BIP0607, Systagenix Wound Management Ltd., Gatwick, UK) was applied on the wound area. Images of the wound area were taken every other day with a scale. The size of wound area at day 12 were analyzed by NIH ImageJ software and expressed as percentage of the wound area on day 0.

## 3. Results

### 3.1. Flavored and Nicotine Containing E-CSE Increased Endothelial Superoxide Production

**Figure 1:**
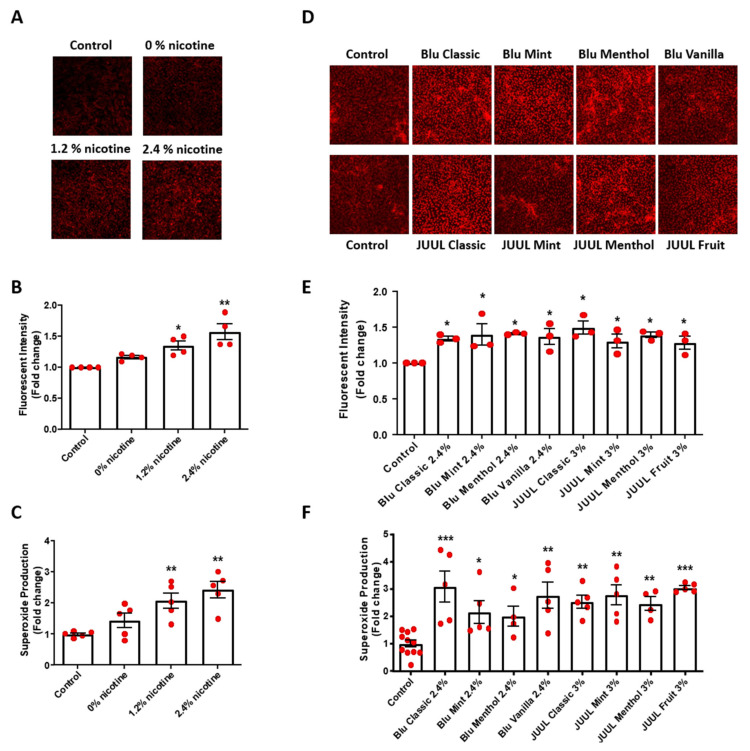
Nicotine-containing and flavored e-cigarettes increase endothelial superoxide production. Confluent bovine aortic endothelial cells (BAECs) were starved overnight and incubated with e-cigarette smoke extract (e-CSE) for 24 h. e-CSEs were prepared from e-cigarettes Gold leaf (Blu) at nicotine levels of 0%, 1.2%, and 2.4%, and flavored e-cigarettes (Blu: Classic, Mint, Menthol, and Vanilla, all with 2.4% nicotine levels; and JUUL: Classic, Mint, Menthol, and Fruit, all with 3% nicotine levels). Treated cells were stained with 2 μmol/L dihydroethidium (DHE) for 30 min. DHE signals from random fields were captured by Nikon Eclipse Ti confocal microscope at 520/610 nm and fluorescent intensity analyzed by NIH ImageJ software. (**A**) Representative DHE images from endothelial cells treated with e-CSE prepared from e-cigarettes containing 0, 1.2%, and 2.4% nicotine levels. (**B**) Grouped DHE imaging data showing dose-dependent increase in superoxide production in cells exposed to e-CSE prepared from e-cigarettes containing 0, 1.2%, and 2.4% nicotine levels. (*n* = 4). (**C**) Superoxide production measurement by electron spin resonance (ESR) using superoxide-specific spin trap CMH (1 mmol/L) showing increased superoxide production in cells exposed to e-CSE prepared from e-cigarettes containing 0, 1.2%, and 2.4% nicotine levels (*n* = 5). (**D**) Representative DHE images from flavored e-CSE-treated BAECs. (**E**) Grouped DHE imaging data showing that flavored e-CSEs increased superoxide production (*n* = 3). (**F**) Superoxide production examined by electron spin resonance (ESR) with superoxide-specific spin trap CMH showing increased superoxide production by flavored e-CSEs (*n* = 5–11). * *p* < 0.05, ** *p* < 0.01, *** *p* < 0.001 vs. Control group by One-Way ANOVA with Newman–Keuls post hoc test.

We examined superoxide production in e-CSE-treated BAECs by dihydroethidium (DHE) fluorescent imaging and electron spin resonance (ESR) as we previously published [15,16,18,19]. As shown in Figure 1, e-CSE increased superoxide production in a nicotine content-dependent manner, determined by both DHE staining (Figure 1A,B) and ESR (Figure 1C). Moreover, e-CSEs prepared from flavored e-cigarettes (Blu: Classic, Mint, Menthol, and Vanilla, all with 2.4% nicotine levels; and JUUL: Classic, Mint, Menthol, and Fruit, all with 3% nicotine levels) also significantly induced superoxide production in endothelial cells (Figure 1D–F). These data indicate robust effects of flavored and nicotine containing e-cigarettes on endothelial superoxide production, suggesting that oxidative stress induced by e-cigarettes likely represents one of the major mechanisms of endothelial damage to contribute to injuries of cardiorespiratory system.

### 3.2. Flavored and Nicotine Containing E-CSE Decreased Endothelial NO Bioavailability

**Figure 2:**
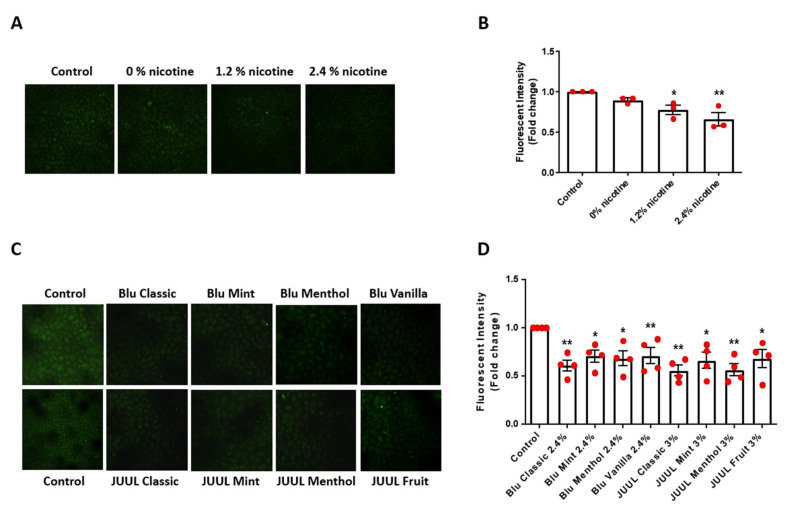
Nicotine-containing and flavored e-cigarettes decrease endothelial NO bioavailability. Confluent bovine aortic endothelial cells (BAECs) were starved overnight and incubated with e-cigarette smoke extract (e-CSE) for 24 h. e-CSEs were prepared from e-cigarette Gold leaf (Blu) at nicotine levels of 0%, 1.2%, and 2.4%, and flavored e-cigarettes (Blu: Classic, Mint, Menthol, and Vanilla, all with 2.4% nicotine levels; and JUUL: Classic, Mint, Menthol, and Fruit, all with 3% nicotine levels). Cells were incubated with nitric oxide (NO)-specific fluorescent probe 4-amino-5-methylamino-2′,7′-difluorofluorescein diacetate (DAF-FM DA, 20 μmol/L) for 1 h. Fluorescent images were captured by Nikon Eclipse Ti confocal microscope at 495/515 nm and fluorescent intensity analyzed by NIH ImageJ software. (**A**) Representative DAF-FM DA images of bioavailable NO levels in endothelial cells treated with e-CSE prepared from e-cigarettes containing different nicotine levels (0, 1.2%, and 2.4%; Gold Leaf). (**B**) Grouped DAF-FM DA imaging data demonstrating nicotine-dependent decline in endothelial NO bioavailability in endothelial cells exposed to e-CSEs prepared from e-cigarettes containing different nicotine levels (*n* = 3). (**C**) Representative DAF-FM DA images of bioavailable NO levels in endothelial cells treated with e-CSEs prepared from flavored e-cigarettes (Blu: Classic, Mint, Menthol, and Vanilla, all with 2.4% nicotine levels; and JUUL: Classic, Mint, Menthol, and Fruit, all with 3% nicotine levels). (**D**) Grouped DAF-FM DA imaging data demonstrating diminished NO bioavailability in endothelial cells exposed to e-CSEs prepared from flavored e-cigarettes. (*n* = 4). * *p* < 0.05, ** *p* < 0.01 vs. Control group by One-Way ANOVA with Newman–Keuls post hoc test.

One of the major consequences of increased superoxide production is oxidative inactivation of NO and consequent endothelial dysfunction [20,21,22,23]. As shown in Figure 2A,B, exposure of endothelial cells to nicotine containing e-CSE resulted in a dose-dependent decrease in bioavailable NO levels determined by DAF-FM fluorescent imaging. Moreover, NO bioavailability was markedly downregulated by flavored e-CSEs (Figure 2C,D). These data indicate that treatment of endothelial cells with nicotine-containing and flavored e-CSEs induces a deficiency in NO bioavailability that is attributed to increased superoxide production, therefore leading to endothelial dysfunction.

### 3.3. Flavored and Nicotine Containing E-CSE Induced Endothelial Cell Apoptosis

**Figure 3:**
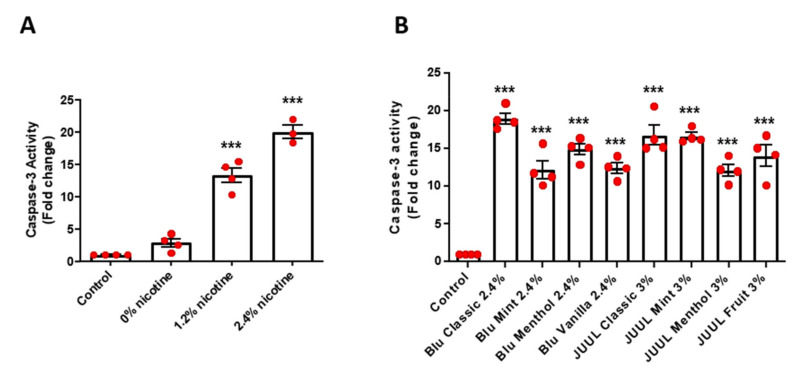
Nicotine-containing and flavored e-cigarettes increase endothelial cell apoptosis. Confluent bovine aortic endothelial cells (BAECs) were starved overnight and incubated with e-cigarette smoke extract (e-CSE) for 24 h. e-CSEs were prepared from e-cigarettes Gold leaf (Blu) at nicotine levels of 0%, 1.2%, and 2.4%, and flavored e-cigarettes (Blu: Classic, Mint, Menthol, and Vanilla, all with 2.4% nicotine levels; and JUUL: Classic, Mint, Menthol, and Fruit, all with 3% nicotine levels). Endothelial cell apoptosis was assessed using an apoptosis assay kit (CASP3F, MilliporeSigma). (**A**) Grouped data of endothelial apoptotic activity from endothelial cells treated with e-CSEs prepared from e-cigarettes containing 0, 1.2%, and 2.4% nicotine levels. (*n* = 4). (**B**) Grouped data of endothelial apoptotic activity from endothelial cells treated with e-CSEs prepared from flavored e-cigarettes (Blu: Classic, Mint, Menthol, and Vanilla, all with 2.4% nicotine levels; and JUUL: Classic, Mint, Menthol, and Fruit, all with 3% nicotine levels). (*n* = 4). *** *p* < 0.001 vs. Control group by One-Way ANOVA with Newman–Keuls post hoc test.

Endothelial cell apoptosis assayed by Caspase 3 activity, known to be induced by oxidative stress, was significantly increased in endothelial cells exposed to e-CSEs prepared from nicotine-containing e-cigarettes in a nicotine content dependent manner (Figure 3A). Endothelial cell apoptosis was also markedly increased by exposure to e-CSEs prepared from various flavored e-cigarettes from two different vendors (Blu classic, Blu Mint, Blu Menthol, and Blu Vanilla, all with 2.5% nicotine; and JUUL classic, JUUL Mint, JUUL Menthol, and JUUL Fruit, all with 3.0% nicotine (Figure 3B).

### 3.4. Flavored and Nicotine Containing E-CSE Impaired Endothelial Cell Wound Healing In Vitro

**Figure 4:**
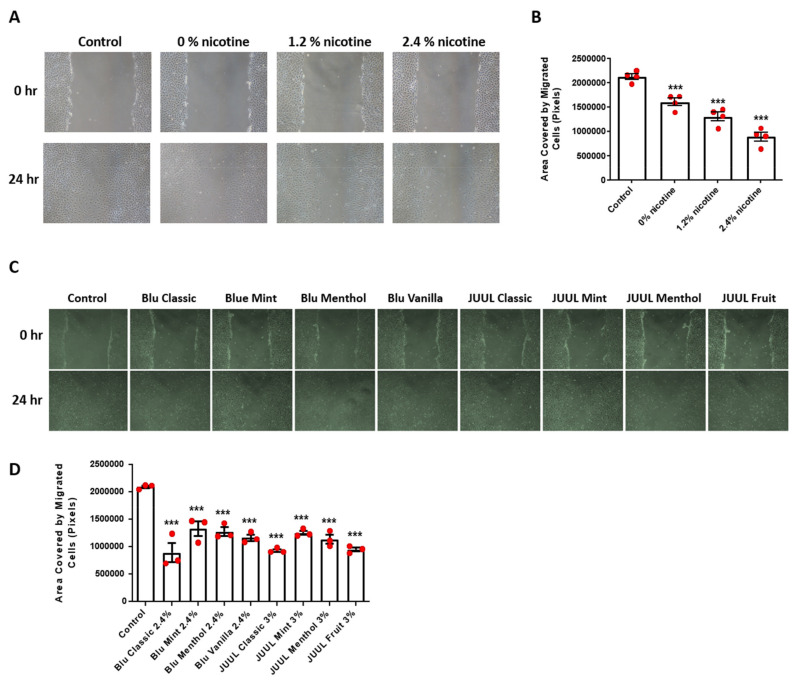
Nicotine containing and flavored e-cigarettes induce impaire endothelial cell wound healing in vitro. Confluent endothelial cells were starved overnight before a 1-mm wound was created using a P1000 tip. The wounded area was labeled and imaged as 0 h, and then exposed to e-CSE. The same area of the wound was imaged again after 24 h. Sizes of the wound areas were analyzed by NIH ImageJ software and presented as area covered by migrated cells (area at 0 h–area at 24 h). (**A**) Representative endothelial monolayer wounds before and after treatment with e-CSEs prepared from e-cigarettes containing different levels of nicotine (Gold leaf [Blu], at nicotine levels of 0%, 1.2%, and 2.4%). (**B**) Grouped wound healing data demonstrating reduced endothelial wound closure in endothelial cells exposed to e-CSEs prepared from e-cigarettes containing different levels of nicotine (*n* = 4). (**C**) Representative endothelial monolayer wounds before and after treatment with e-CSEs prepared from flavored e-cigarettes (Blu: Classic, Mint, Menthol, and Vanilla, all with 2.4% nicotine levels; and JUUL: Classic, Mint, Menthol, and Fruit, all with 3% nicotine levels). (**D**) Grouped would healing data demonstrating reduced endothelial wound closure in endothelial cells exposed to e-CSEs prepared from flavored e-cigarettes (*n* = 3). *** *p* < 0.001 vs. Control group by One-Way ANOVA with Newman–Keuls post hoc test.

To examine effects of e-CSE on angiogenesis in vitro, we performed endothelial cell monolayer wound assay. Of note, endothelial monolayer wound closure was substantially inhibited by e-CSE in a nicotine dependent manner (Figure 4A,B). In addition, exposure of wounded endothelial monolayers to e-CSEs prepared from flavored e-cigarettes also resulted in impaired monolayer wound closure (Figure 4C,D). These data indicate that coordinated endothelial proliferation and migration is injured by treatment with e-CSEs prepared from flavored and nicotine-containing e-cigarettes, leading to impaired angiogenesis.

### 3.5. Flavored and Nicotine Containing E-CSE Induced Impaired Diabetic Wound Healing In Vivo

**Figure 5:**
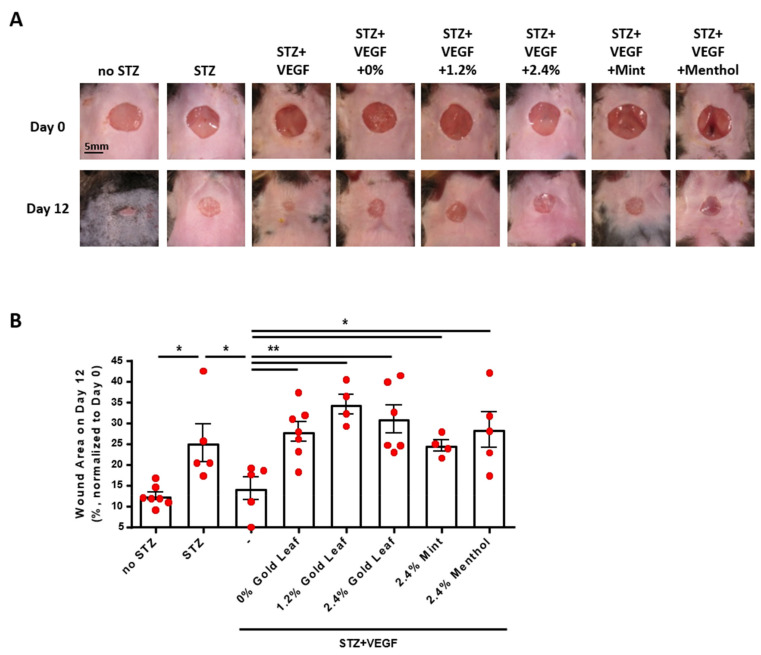
Nicotine-containing and flavored e-cigarettes induce impaired diabetic wound healing in vivo. Wild type male C57BL/6 mice (10–12 weeks old) were treated with two injections (i.p.) of streptozocin (STZ, 100 mg/kg). Seven days after diabetic induction, a 6-mm skin wound was made by a biopsy punch, and the wound was imaged and labeled as Day 0. The wound area was treated with VEGF (200 ng) in the presence or absence of e-CSE (no dilution from 100% stock of original preparation, 5 μL) every other day till Day 12. Scale: 5 mm. (**A**) Representative images of wound area on Day 0 and Day 12 in diabetic mice treated with e-CSEs prepared from e-cigarettes containing different levels of nicotine (Gold leaf [Blu] with 0%, 1.2%, and 2.4% nicotine levels) and different flavors (Mint and Menthol with 2.4% nicotine from Blu). (**B**) Grouped data for sizes of wound areas on Day 12 as a percentage of wound area on Day 0, indicating delayed diabetic wound healing in mice treated with e-CSEs prepared from e-cigarettes containing different levels of nicotine and different flavors (*n* = 4–7). * *p* < 0.05, ** *p* < 0.01 by One-Way ANOVA with Newman–Keuls post hoc test.

Vascular endothelial growth factor (VEGF) is a potent angiogenic protein, downregulation of which and its receptor has been implicated in defective diabetic wound healing [19]. We next examined effects of e-cigarettes on VEGF-dependent diabetic wound healing. Diabetes was induced in wild type C57BL/6 mice by injection (i.p.) of streptozotocin (STZ, 100 mg/kg), increasing blood glucose levels from 156.4 ± 6.10 mg/dL in the control mice (*n* = 7) to 371.1 ± 14.5 mg/dL in the diabetic mice (*n* = 36). Wounds were created on the neck skin of the mice, and wound closure monitored as we previously published [19] and described in the Methods section. In STZ-induced diabetic mice, delayed wound closure was fully restored by supplementation of VEGF, whereas VEGF-driven wound closure was completely inhibited by e-CSEs prepared from Blu Gold leaf e-cigarettes with 0%, 1.2%, and 2.4% nicotine levels, and from flavored Blu e-cigarettes of 2.4% Mint and 2.4% Menthol (Figure 5A,B). Among the different flavors tested, gold leaf flavor seemed to show the most delayed wound healing in response to VEGF (Figure 5A,B). Therefore, our data, for the first time, demonstrate that VEGF-dependent wound healing is impaired by e-cigarettes in diabetic mice in vivo, indicating that regulation of e-cigarette use should be enhanced in diabetic/surgical patients to avoid deteriorating consequences of impaired wound healing.

## 4. Discussion

In the present study, we elucidate novel molecular mechanisms underlying adverse effects of e-cigarettes on endothelial cells, highlighting impaired angiogenesis and impaired diabetic wound healing. Oxidative stress has been shown to play a crucial role in endothelial dysfunction and consequent cardiovascular risk associated with cigarette smoking, while the effects and mechanisms of e-cigarette vaping on endothelial function had remained unclear until our present study, which revealed increased ROS production, decreased NO bioavailability, and increased endothelial cell apoptosis by exposure to e-CSEs prepared from nicotine containing and flavored e-cigarettes. We believe these features of endothelial dysfunction underlie impaired angiogenesis and diminished wound healing under diabetic environment.

**Figure 6:**
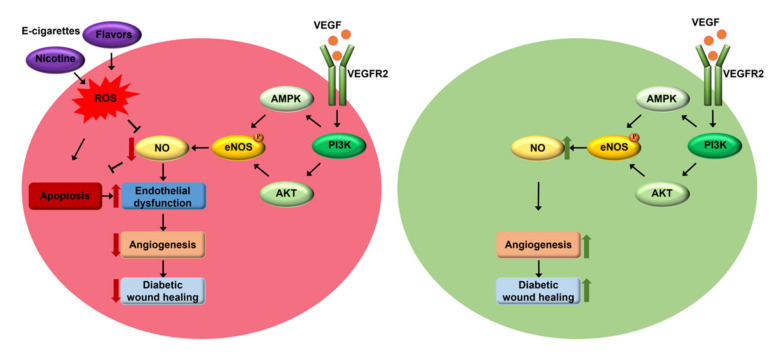
Molecular mechanisms underlying e-cigarette induced impairment in angiogenesis and diabetic wound healing. Under normal conditions, vascular endothelial growth factor (VEGF) activates PI3K dependent eNOS phosphorylation via AKT and AMPK to result in elevated NO production to mediate enhanced angiogenesis and improved diabetic wound healing. Exposure to nicotine-containing or flavored e-cigarettes results in increased reactive oxygen species (ROS) production, reduced NO bioavailability and endothelial cell apoptosis, leading to endothelial dysfunction and consequent deficiencies in angiogenesis and diabetic wound healing.

Our data indicate that treatment of endothelial cells with e-CSEs prepared from flavored and nicotine containing e-cigarettes induced marked deficiency in NO bioavailability attributed to increased superoxide production defined by both fluorescent imaging and ESR analyses. In addition, endothelial cell apoptosis was increased by e-CSEs prepared from flavored and nicotine containing e-cigarettes, which is consistent with earlier findings that induced pluripotent stem cells-derived endothelial cells (iPSC-ECs) exerted reduced NO production and increased apoptosis in response to e-cigarette vapor [24]. Since the flavored e-cigarettes we used for the present study all contain nicotine from the two major vendors with nicotine-free products not available, potential interactions between nicotine and flavoring additives need to be further investigated. Moreover, wound closure reflecting the coordinated action of endothelial proliferation and migration was impaired by exposure to flavored and nicotine containing e-CSEs in endothelial cells. Collectively, these results indicate that vasculature rich organs might be prone to e-cigarette vaping induced injuries via increased oxidative stress, deficiency in NO bioavailability, and endothelial toxicity, all of which are known to contribute to endothelial dysfunction. Of note, VEGF facilitated repairs of incisional skin wound in diabetic mice were completely blocked by flavored and nicotine containing e-CSE treatments. Given that NO is a critical mediator of VEGF dependent angiogenesis [21], and that NO enhances VEGF synthesis, e-cigarette induced deficiency in NO bioavailability due to increased ROS production represents the central mechanism underlying impaired wound healing in diabetic environment. These novel findings are schematically summarized in Figure 6.

Therefore, our findings provide novel insights into e-cigarette use contribution to chronic diabetic peripheral tissue damages, by worsening of vasculopathy through ROS dependent NO deficiency resulting in a delay in wound healing.

## 5. Conclusions

In conclusion, our results strongly indicate that e-cigarette use should be prohibited in patients with diabetes, or in surgical patients, to prevent aggravated peripheral tissue damages from impaired angiogenesis and wound healing. Rigorous regulations on e-cigarette use, therefore, are considered particularly important for diabetic and/or surgical patients.

## Data Availability

The data presented in this study are available on reasonable request from the corresponding author.

## References

[1] Hajek P., Phillips-Waller A., Przulj D., Pesola F., Myers Smith K., Bisal N., Li J., Parrott S., Sasieni P., Dawkins L. (2019). A Randomized Trial of E-Cigarettes versus Nicotine-Replacement Therapy. N. Engl. J. Med..

[2] WHO (2021). https://www.who.int/teams/health-promotion/tobacco-control/global-tobacco-report-2021.

[3] Cai H., Garcia J.G.N., Wang C. (2020). More to Add to E-Cigarette Regulations: Unified Approaches. Chest.

[4] Park-Lee E., Ren C., Sawdey M.D., Gentzke A.S., Cornelius M., Jamal A., Cullen K.A. (2021). Notes from the Field: E-Cigarette Use Among Middle and High School Students—National Youth Tobacco Survey, United States, 2021. MMWR Morb. Mortal. Wkly. Rep..

[5] Cai H., Wang C. (2017). Graphical review: The redox dark side of e-cigarettes; exposure to oxidants and public health concerns. Redox Biol.

[6] CDC (2021). https://www.cdc.gov/tobacco/basic_information/e-cigarettes/severe-lung-disease.html#epi-chart.

[7] Reagan-Steiner S., Gary J., Matkovic E., Ritter J.M., Shieh W.J., Martines R.B., Werner A.K., Lynfield R., Holzbauer S., Bullock H. (2020). Pathological findings in suspected cases of e-cigarette, or vaping, product use-associated lung injury (EVALI): A case series. Lancet Respir. Med..

[8] Chung S., Baumlin N., Dennis J.S., Moore R., Salathe S.F., Whitney P.L., Sabater J., Abraham W.M., Kim M.D., Salathe M. (2019). Electronic Cigarette Vapor with Nicotine Causes Airway Mucociliary Dysfunction Preferentially via TRPA1 Receptors. Am. J. Respir. Crit. Care Med..

[9] Madison M.C., Landers C.T., Gu B.H., Chang C.Y., Tung H.Y., You R., Hong M.J., Baghaei N., Song L.Z., Porter P. (2019). Electronic cigarettes disrupt lung lipid homeostasis and innate immunity independent of nicotine. J. Clin. Investig..

[10] Gonzalez J.E., Cooke W.H. (2021). Acute effects of electronic cigarettes on arterial pressure and peripheral sympathetic activity in young nonsmokers. Am. J. Physiol. Heart Circ. Physiol..

[11] Moheimani R.S., Bhetraratana M., Peters K.M., Yang B.K., Yin F., Gornbein J., Araujo J.A., Middlekauff H.R. (2017). Sympathomimetic Effects of Acute E-Cigarette Use: Role of Nicotine and Non-Nicotine Constituents. J. Am. Heart Assoc..

[12] El-Mahdy M.A., Mahgoup E.M., Ewees M.G., Eid M.S., Abdelghany T.M., Zweier J.L. (2021). Long-term electronic cigarette exposure induces cardiovascular dysfunction similar to tobacco cigarettes: Role of nicotine and exposure duration. Am. J. Physiol. Heart Circ. Physiol..

[13] Nguyen A., Cai H. (2006). Netrin-1 induces angiogenesis via a DCC-dependent ERK1/2-eNOS feed-forward mechanism. Proc. Natl. Acad. Sci. USA.

[14] Li H., Li Q., Zhang Y., Liu W., Gu B., Narumi T., Siu K.L., Youn J.Y., Liu P., Yang X. (2019). Novel Treatment of Hypertension by Specifically Targeting E2F for Restoration of Endothelial Dihydrofolate Reductase and eNOS Function Under Oxidative Stress. Hypertension.

[15] Chalupsky K., Cai H. (2005). Endothelial dihydrofolate reductase: Critical for nitric oxide bioavailability and role in angiotensin II uncoupling of endothelial nitric oxide synthase. Proc. Natl. Acad. Sci. USA.

[16] Zhang Y., Li Q., Youn J.Y., Cai H. (2017). Protein Phosphotyrosine Phosphatase 1B (PTP1B) in Calpain-dependent Feedback Regulation of Vascular Endothelial Growth Factor Receptor (VEGFR2) in Endothelial Cells: Implications in vegf-dependent angiogenesis and diabetic wound healing. J. Biol. Chem..

[17] Youn J.Y., Nguyen A., Cai H. (2012). Inhibition of XO or NOX attenuates diethylstilbestrol-induced endothelial nitric oxide deficiency without affecting its effects on LNCaP cell invasion and apoptosis. Clin. Sci. (Lond.).

[18] Siu K.L., Gao L., Cai H. (2016). Differential Roles of Protein Complexes NOX1-NOXO1 and NOX2-p47phox in Mediating Endothelial Redox Responses to Oscillatory and Unidirectional Laminar Shear Stress. J. Biolog. Chem..

[19] Oak J.H., Cai H. (2007). Attenuation of angiotensin II signaling recouples eNOS and inhibits nonendothelial NOX activity in diabetic mice. Diabetes.

[20] Cai H., Harrison D.G. (2000). Endothelial dysfunction in cardiovascular diseases: The role of oxidant stress. Circ. Res..

[21] Cai H. (2005). NAD(P)H oxidase-dependent self-propagation of hydrogen peroxide and vascular disease. Circ. Res..

[22] Cai H. (2005). Hydrogen peroxide regulation of endothelial function: Origins, mechanisms, and consequences. Cardiovasc. Res..

[23] Zhang Y., Murugesan P., Huang K., Cai H. (2020). NADPH oxidases and oxidase crosstalk in cardiovascular diseases: Novel therapeutic targets. Nat. Rev. Cardiol..

[24] Lee W.H., Ong S.G., Zhou Y., Tian L., Bae H.R., Baker N., Whitlatch A., Mohammadi L., Guo H., Nadeau K.C. (2019). Modeling Cardiovascular Risks of E-Cigarettes With Human-Induced Pluripotent Stem Cell-Derived Endothelial Cells. J. Am. Coll. Cardiol..

